# Generalized Lymphatic Anomaly and Gorham–Stout Disease: Overview and Recent Insights

**DOI:** 10.1089/wound.2018.0850

**Published:** 2019-06-06

**Authors:** Michio Ozeki, Toshiyuki Fukao

**Affiliations:** Department of Pediatrics, Gifu University Graduate School of Medicine, Gifu, Japan.

**Keywords:** lymphatic malformation, complex lymphatic anomaly, kaposiform lymphangiomatosis, central conducting lymphatic anomaly, osteolysis

## Abstract

**Significance:** Generalized lymphatic anomaly and Gorham–Stout disease are extremely rare diseases with severe symptoms and poor prognosis. The etiology and clinical presentation of the patients remain poorly defined, but recent research has attempted to determine the pathogenesis of these diseases.

**Recent Advances:** In recent years, the characteristics of complex lymphatic anomalies have been revealed. Kaposiform lymphangiomatosis is recognized as a new entity that has an aggressive course and poor prognosis. Genetic analysis revealed somatic mutations in genes associated with the phosphoinositide 3-kinase (PI3K) pathway in lymphatic malformation lesions. Somatic *NRAS* mutation in lymphatic endothelium from a generalized lymphatic anomaly patient was also detected as a potential cause of disease. Furthermore, studies demonstrated the efficacy of the mammalian target of rapamycin (mTOR) inhibitor sirolimus for these lymphatic diseases.

**Critical Issues:** These diseases have overlapping symptoms, imaging features, and complications, leading to difficulty in their differential diagnosis. In addition, there are no standard therapies. Therefore, we need to determine the differences among these diseases to not only diagnose but also treat them appropriately.

**Future Directions:** Further investigations should reveal differences in the clinical features and findings of radiological, pathological, and genetic examinations to manage each disease appropriately. Somatic mutation in genes encoding RAS/PI3K/mTOR signaling pathway components could be associated with the pathogenesis of these diseases and may be novel targets for drug therapies.

**Figure:**
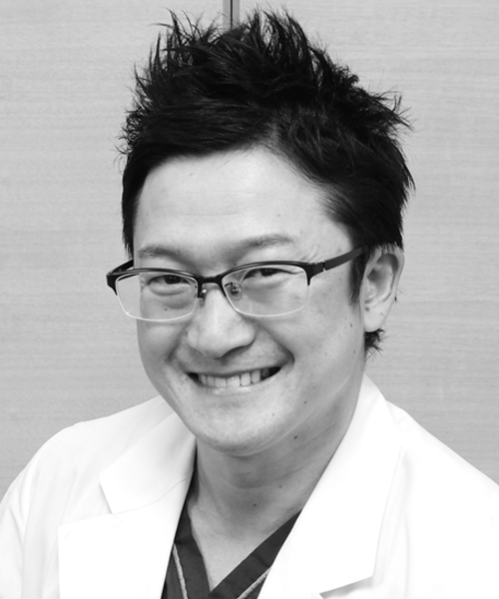
Michio Ozeki, MD, PhD

## Scope and Significance

Generalized lymphatic anomaly^1–3^ (GLA), Gorham–Stout disease^4,5^ (GSD), Kaposiform lymphangiomatosis^6–8^ (KLA), and channel-type lymphatic malformations (LMs) are classified according to their pathology^1^ (Table 1); however, some disease forms with differing pathologies are not separately defined entities.^9^ Furthermore, central conducting lymphatic anomaly (CCLA), a channel-type LM^1,2^ with unclear etiology, might represent a spectrum of lymphatic pathologies. Because clinical findings overlap, and disease etiology, management, outcomes, and sequelae are unclear, intractable LMs (termed complex lymphatic anomalies [CLAs])^2^ are difficult to diagnose. We review recent studies reporting different clinical characteristics of vascular anomalies to further understand their pathogenesis and optimal treatment.

**Table 1.** International society for the study of vascular anomalies classification of vascular anomalies

Simple vascular malformations Va
LMs
Common (cystic) LM PIK3CA^a^
Macrocystic LM
Microcystic LM
Mixed cystic LM
GLA
KLA
LM in GSD
Channel-type LM, (CCLA, lymphangiectasia)
“Acquired” progressive lymphatic anomaly (so-called acquired progressive “lymphangioma”)
Primary lymphedema
Others

^a^ The causative gene.

CCLA, central conducting lymphatic anomaly; GLA, generalized lymphatic anomaly; GSD, Gorham–Stout disease; KLA, kaposiform lymphangiomatosis; LMs, lymphatic malformations; PIK3CA, phosphatidylinositol-4,5-bisphosphate 3-kinase catalytic subunit alpha.

## Translational Relevance

Congenital structural abnormalities of lymphatic vessels and the thoracic duct, or abnormal proliferation of local lymphatic endothelial cells may cause the abnormal conditions in these patients. Vascular endothelial growth factor (VEGF)-C can induce controlled lymphatic recovery after injury.^10^ Thus, VEGF-C pathological models are important for understanding the pathogenesis of CLAs.^11–13^ Recent studies have investigated lymphangiogenesis and lymphatic vasculature remodeling and suggested that genetic abnormalities in the RAS/mitogen-activated protein (MAPK) and phosphoinositide 3-kinase (PI3K)/mammalian target of rapamycin (mTOR) signaling pathways could be responsible for these lymphatic diseases.^14–18^

## Clinical Relevance

**Figure 1.**
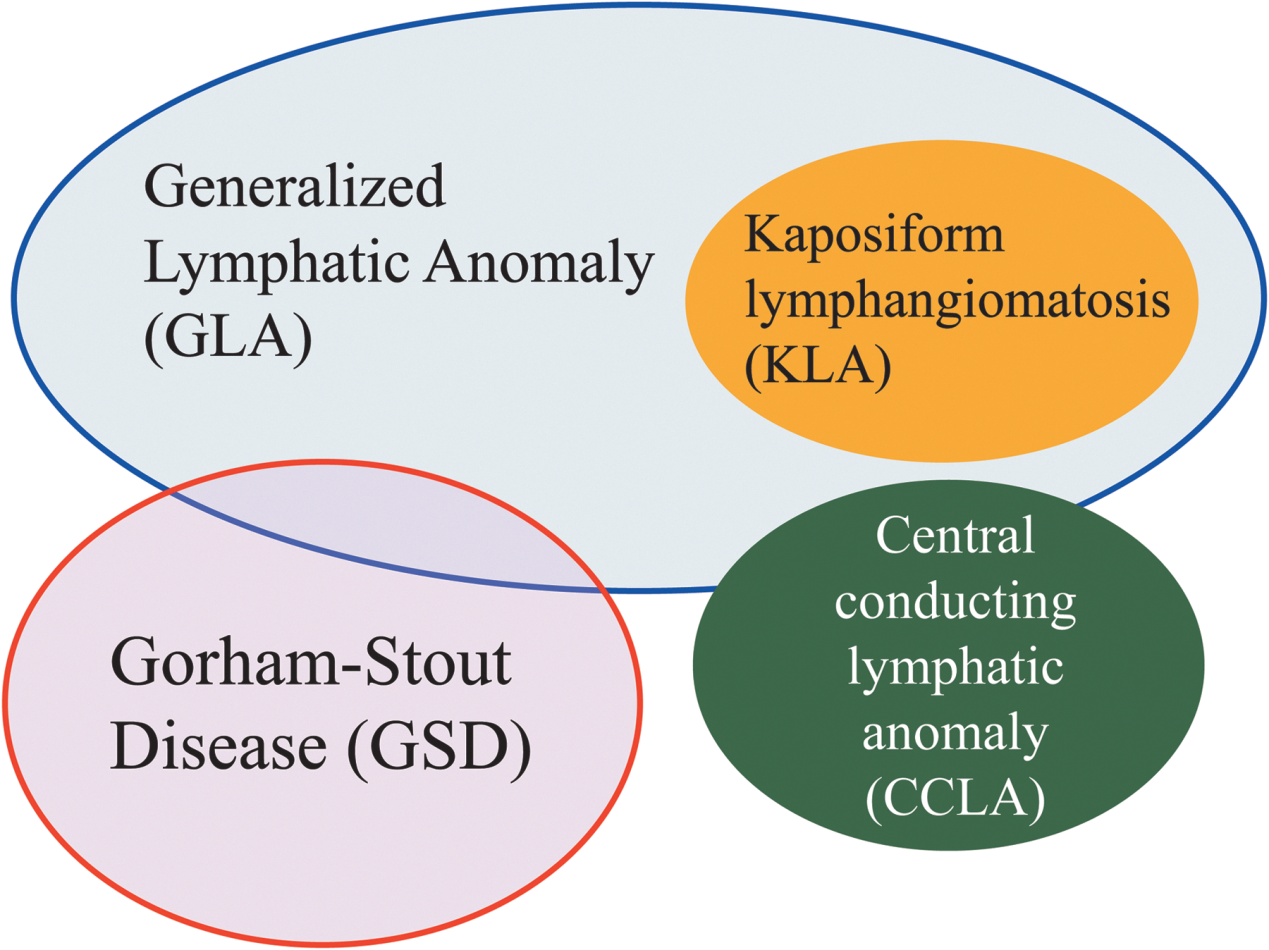
Overlapping features of complex lymphatic anomalies. Complex lymphatic anomalies are overlapping their clinical symptoms and characteristics.

CLAs involve multiple organs and have diverse symptoms, but an appropriate diagnosis is difficult as their clinical findings overlap (Fig. 1). Notably, osteolytic lesions are useful for distinguishing between GLA/KLA and GSD.^19^ Patients with KLA have a severe coagulation disorder and life-threatening hemorrhagic pericardial and pleural effusion.^20^ The histological hallmark of KLA is kaposiform, hemosiderotic, spindle-shaped lymphatic endothelial cells arranged in clusters.^6^ Intranodal lymphangiography and dynamic contrast-enhanced magnetic resonance lymphangiography (DCMRL) are new imaging techniques able to detect the anatomical findings of the thoracic duct and lymphatic flow, which can lead to a correct diagnosis of CCLA.^21^

## Discussion of Findings and Relevant Literature

### LMs in International society for the study of vascular anomalies classification

In 1996, the first International Society for the Study of Vascular Anomalies (ISSVA) classification was approved at the 11th workshop. In 2014, an updated classification was approved at the 20th workshop. This classification has been promoted to spread correct understanding of vascular anomalies. When clinicians and scientists plan their clinical and basic research, they often confuse inconsistent terminology. The correction and simplification of that terminology and systematization of guidelines have promoted a more uniform approach to researching vascular anomalies. Common (cystic) LMs (macrocystic, microcystic, or mixed) are the most common lymphatic anomalies that occur in infants. These lesions are solitary and cystic-appearing soft-tissue masses that commonly arise in neck, mediastinum, and retroperitoneum. Their typical appearance and symptoms help us to differentiate them from CLAs. The updated classification categorized GLA and GSD as separated systemic LMs.^22^ GLA is a rare multiorgan disease involving organs such as lung, mediastinum, spleen, and proliferating lymphatic vessels diffusely. Its thoracic involvement can cause respiratory failure and is associated with poor prognosis. This condition predominantly affects children and adolescence and has variable symptoms. GSD is a rare and enigmatic disease characterized by the presence of an intraosseous LM. The osteolysis progresses destructively with the developing and proliferating LM. In the World Health Organization classification, KLA was distinguished as a rare tumor of lymphatic vessel origin.^23^ However, KLA was defined as a provisionally unclassified vascular anomaly in the updated ISSVA classification of 2014 because KLA patients have invasive and aggressive features, but the pathological findings show a hypoproliferative rate. KLA is described as an aggressive disease of the lymphatic system and has foci of “kaposiform” abnormal spindle lymphatic endothelial cells.^6–8^ In a review from 2000, Faul described how some patients with a GLA called diffuse pulmonary lymphangiomatosis showed the presence of spindle-shaped cells around endothelial cells, which contrasted with primary pulmonary lymphangiectasia,^9^ but this was not designated as a specific disease entity. Both GLA and KLA patients have multiple organs affected and the lesions can cause similar symptoms such as pleural effusion, ascites, and skin involvements. In the 2018 updated ISSVA classification, KLA was categorized as a subtype of GLA^1^ (Table 1). In contrast, channel-type LM, CCLA, previously called lymphangiectasia, is characterized by dilated lymphatic channels, dyskinesia, or obstructive impairment. The abnormalities of lymphatic structure can induce problem of clearance of lymph.^2^ The symptoms depend on the site of the anomaly, such as primary intestinal lymphangiectasia, pulmonary lymphangiectasia, which causes protein-losing enteropathy, pleural effusion, peripheral lymphedema, and lymphorrhea. Primary lymphedema is sporadic, or rarely familial lymphatic malformative disease. It mainly involves the lower extremities and the lymphatic subcutaneous fibroadipose lesions are not curable and grow gradually. It has been determined that most types of primary lymphedema are caused by various genetic mutations.^1^ Against this background, in some patients, it is difficult to achieve a correct diagnosis of CLAs because the clinical findings overlap among these conditions. There has been a lack of information on not only their etiology but also their specific management, long-term outcomes, and disease sequelae. However, recent studies on the spectrum of CLAs have suggested that there are significant differences in their clinical characteristics. Further study should be performed, leading to a more comprehensive understanding of the etiology and optimal treatment for CLAs.

### Etiology and genetics

#### The causative genes of LM

**Figure 2.**
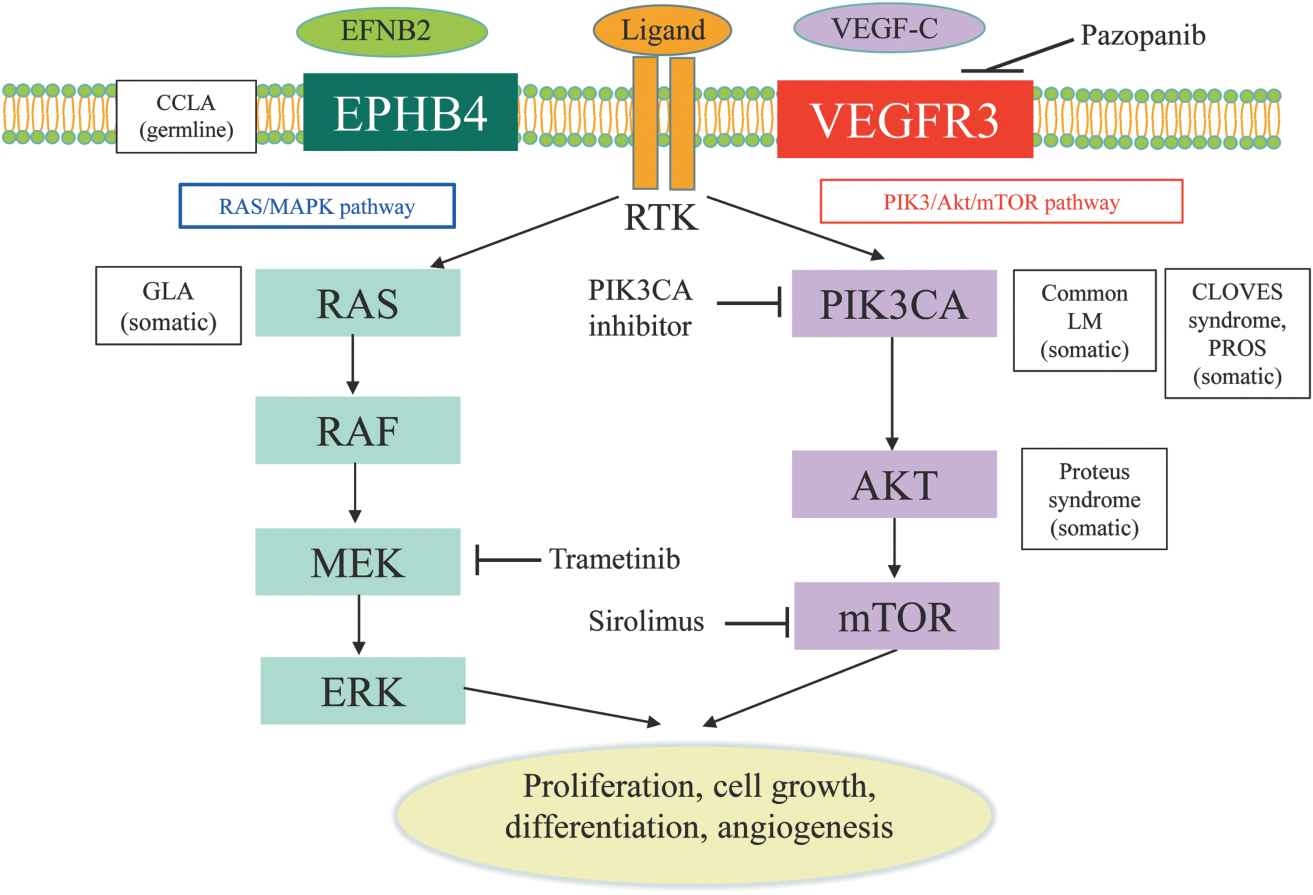
Mutations and signaling pathways involved in LMs. Mutations in *EPHB4* and *NRAS* lead to activate the signaling of RAS/MEK/ERK pathway. Mutations in *PIK3CA* and *AKT* lead to activate the signaling of PIK3/AKT/mTOR pathway. CLOVES, congenital lipomatous overgrowth, vascular malformations, epidermal nevi, scoliosis/skeletal and spinal; EFNB2, ephrin-B2; EPHB4, ephrin B4; GLA, generalized lymphatic anomaly; LM, lymphatic malformation; mTOR, mammalian target of rapamycin; PIK3CA, phosphatidylinositol-4,5-bisphosphate 3-kinase catalytic subunit alpha; PROS, PIK3CA-related overgrowth spectrum; RTK, receptor tyrosine kinase; VEGF-C, vascular endothelial growth factor-C; VEGFR, VEGF receptor.

The development of next-generation sequencing has identified low-level somatic mutations in sporadic lesions of vascular anomalies,^15^ and the updated ISSVA classification now contains a list of causal genes.^1^ There are over 40 genes that are associated with the etiology of vascular anomalies. Although primary lymphedemas are caused by germline mutation, LMs are sporadic, not familial, and caused by somatic changes in components of the PI3K/AKT/mTOR pathway (Fig. 2). Mutations in *PIK3CA* have been found in 16 of 17 specimens of cystic LM (mutant allele frequency, 0.8% to 10%).^24^ Five PIK3CA (phosphatidylinositol-4,5-bisphosphate 3-kinase catalytic subunit alpha) mutations (p.E542K, p.H1047R, p.C420R, p.E545K, and p.H1047L) were detected in patients with LM. However, patients with other vascular malformative/overgrowth disorders also had the same mutations. Another report investigated isolated lymphatic endothelial cells from a patient who had the angiogenic phenotype. The authors identified two mutations in these lymphatic endothelial cells that showed higher proliferation and AKT activation than those of human lymphatic endothelial cells.^17^ These features can form part of a syndrome such as congenital lipomatous overgrowth, vascular malformations, epidermal nevi, scoliosis/skeletal and spinal (CLOVES) syndrome, Proteus syndrome, and Klippel–Trenaunay syndrome, which feature lymphatic disruption and overgrowth. Recent studies have shown that PIK3CA-related overgrowth spectrum (PROS) is caused by somatic mosaicism of variants in genes of the PI3K pathway.^25^ PROS includes hemihyperplasia multiple lipomatosis, CLOVES syndrome, macrodactyly, fibroadipose hyperplasia or overgrowth, Klippel–Trenaunay syndrome, megalencephaly–capillary malformation (MCAP or M-CM), fibroadipose infiltrating lipomatosis/facial infiltrative lipomatosis, and dysplastic megalencephaly.^1^ Although the terms used to describe vascular anomalies have been made more scientific by the ISSVA based on histopathological findings, differentiation of these diseases is challenging based on their phenotypic presentation alone because patients within this spectrum of overgrowth syndromes have overlapping clinical features.

Regarding other CLAs, little has been reported on the associated genetic abnormalities. However, Manevitz-Mendelson *et al.* reported the possibility that somatic *NRAS* mutation causes GLA.^18^ A variety of human malignancies have activating mutations in *RAS* proto-oncogenes (*KRAS*, *HRAS*, and *NRAS*), which have an important role in carcinogenesis.^26^ Manevitz-Mendelson *et al.* analyzed lymphangiomatosis endothelial cells (LyECs), which were isolated from a GLA patient using CD31-coated magnetic beads. A somatic mutation in *NRAS* gene (c.182A >G, Q61R) in fewer than 30% of the alleles was identified in LyECs.^18^ In addition, the *NRAS* mutation plays key roles in the regulation of angiogenesis and lymphangiogenesis. Treatment with an mTOR inhibitor, sirolimus, and an MEK inhibitor, trametinib, had an effect of reducing the viability of the LyECs through inhibition of the phosphorylation of AKT and ERK, and so might be a novel target treatment of GLA. Furthermore, another group found that CCLA might be associated with a germline mutation in *EPHB4.*^27^ Ephrin B4 (EPHB4) encodes ephrin B-type receptor 4 that binds to ephrin B2 (EFNB2). The EFNB2/EPHB4 pathway plays a role in the determination of venous and lymphatic cell fate and lymphatic valve development.^28,29^ In this study, the zebrafish model of *EPHB4* mutation was shown to mimic the lymphatic presentation of CCLA, including the abnormality of lymphatic vessel branching and formation. The model demonstrated that this mutation might be responsible for the differentiation defects of lymphatic vessels in CCLA patients. This can also be effectively reduced by treatment with sirolimus and trametinib. These studies demonstrated that these genes can cause the pathogenic etiology of these diseases and the inhibition of these genes might be a target for treatment.

#### The mechanisms of osteolysis in GSD

**Figure 3.**
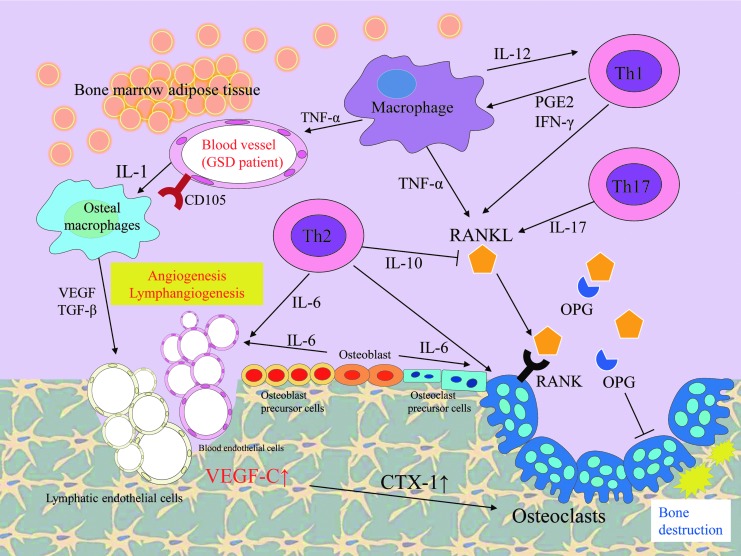
Schematic diagram summarizing the possible pathway of key elements in GSD. In this schema, immunological responses, related secreted cytokines, and cells are shown. The *arrows* indicate the activated directions and lateral lines indicate suppressive actions. The development of lesions in GSD has been associated with angiogenic and lymphangiogenic factors. The formation of bone lymphatics and osteoclast-mediated bone resorption can be seen in affected bone lesions of GSD patients. The endothelial cells in GSD patients highly express CD105 and activate osteal macrophages by producing IL-1. VEGF and TGF-β produced by osteal macrophages promote angiogenesis and lymphangiomatosis. CTX-1, C-terminal telopeptide of type I collagen; GSD, Gorham–Stout disease; IFN, interferon; IL, interleukin; OPG, osteoprotegerin; PGE2, prostaglandin E2; RANK, receptor activator of nuclear factor κB; RANKL, ligand for receptor activator of nuclear factor κB; TGF, transforming growth factor; Th, helper T; TNF, tumor necrosis factor.

**Figure 4.**
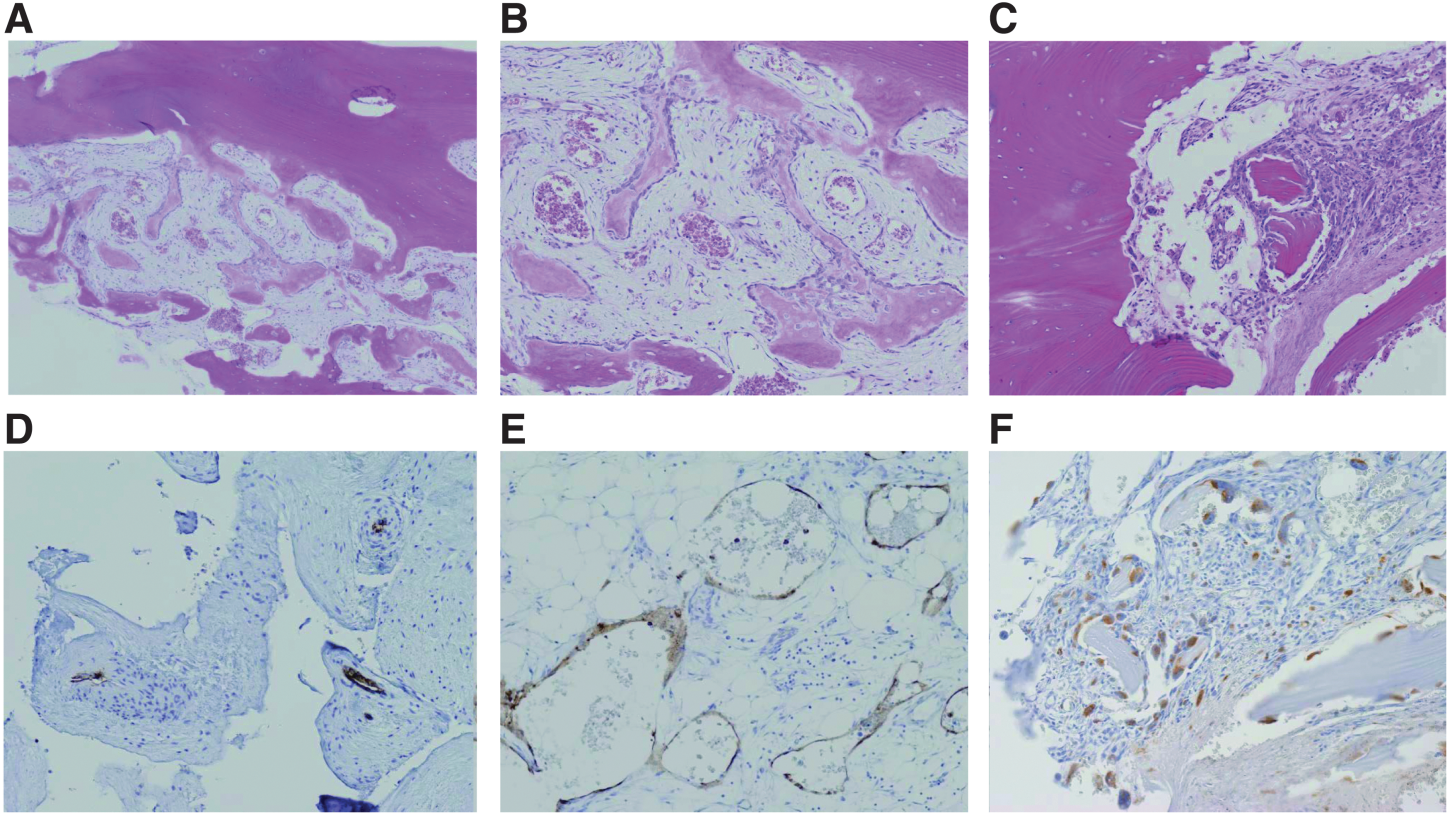
Pathological findings in patients with GSD. In the pathological examinations of GSD patients, diffuse lymphatic vessel proliferation with progressive osteolysis and the activation of osteoclasts are the characteristic features. **(A–F)** A 19-year-old male with GSD. The patient suffered from pain of the right femur, for which no specific cause was evident. **(A–C)** The typical findings of affected bone are activated osteoclasts and thin-walled endothelium-lined capillaries of vascular or lymphatic origin **(A**, low-power field; **B** and **C**, high-power field; H&E stain). **(D)** CD31 immunostaining delineates the vascular endothelium. **(E)** D2-40 immunostaining was positive as the endothelium of lymphatic channels. **(F)** CD68 immunostaining delineates the increases in monocytes and osteoclasts at the affected osteolytic lesion. H&E, hematoxylin and eosin.

The mechanisms behind the osteolysis in GSD are unknown, but various hypotheses have been proposed. It is known that osteolytic lesion in GSD is associated with local proliferation of lymphatic vessels.^30^ The activation of osteoclasts and lymphangiogenesis are essential for the development of GSD and are repeatedly observed in its lesions (Figs. 3 and 4). Osteolysis is also seen in GLA and KLA, but these osteolytic lesions consist of diffuse dilated lymphatic endothelial cells. Therefore, the etiology of GLA and KLA is distinct from that of GSD. Although, lymphatic vessels are abnormally distributed in affected bone lesions in GSD, and normal bone shows an absence of such vessels. The mechanism and triggers of invasion of lymphatic endothelial cells into bone remain unknown. It is considered that activation of the osteoclastogenic process is induced by immune mechanisms in affected bone tissue because the number of osteoclasts increases in the GSD lesion.^31^ Alternatively, osteolysis could be caused by the abnormal proliferation of lymphatic vessels compressing bone. Moreover, the activity of osteoclasts is influenced by some cytokines that are secreted by the lymphatic endothelial cells. The endothelial cells express VEGF receptors (VEGFRs) and VEGF signaling plays an important role in angiogenesis and lymphangiogenesis.^32^ The receptor for VEGF-C/D, VEGFR-3, is a key regulator of lymphangiogenesis.^33^ A high local concentration of VEGF-C or other prolymphangiogenic factors (VEGF-A, VEGF-D, and angiopoietin) can induce angiogenesis and lymphangiogenesis in the bone marrow of patients with GSD. In fact, VEGF-C level is increased in the serum of patients.^34^ A recent study also revealed that the transgenic overexpression of VEGF-C in bone stimulates the formation of bone lymphatics and osteoclast-mediated bone resorption that resembles GSD.^13^ Circulating levels of C-terminal telopeptide of type I collagen (CTX-1), a small peptide generated by osteoclast-mediated cleavage of collagen I,^35^ were found to be significantly higher and the number and activity of osteoclasts were increased in VEGF-C transgenic mice. Furthermore, osteal macrophages and activated T cells secrete various cytokines and promote angiogenesis and lymphangiomatosis (Fig. 3). Activated T cells produce the ligand for receptor activator of nuclear factor κB (RANK) and osteoprotegerin (OPG) binds to ligand for RANK (RANKL), which inhibit interaction with RANK and osteoclastic differentiation and activation.^36^ Type 2 T helper cells, osteoblasts, and dendritic cells produce interleukin (IL)-6 and can promote the angiogenesis and differentiation of osteoclasts. IL-6 increases bone resorption through the RANKL induction of mesenchymal cells. The recent study reported by Hominick *et al*.^13^ revealed a crucial point, but it is unclear why the local level of VEGF-C increases and triggers abnormal angiogenesis and lymphangiogenesis at GSD lesions. An important issue in this context is what induces the immunological triggering of osteal macrophage and endothelial cell activation. This might be associated with somatic gene mutation or inopportune function of bone-resident cells. To clarify this, further investigations should be performed using affected tissue samples. The main factors involved in this triggering may be an appropriate and effective target for the drug treatment of GSD.

### Clinical features and differential diagnosis of CLAs

The characteristics of CLAs and differential features from other diseases are shown in Table 2. CLAs involve multiple organs, have diverse symptoms, and are common in children and adolescents. One report described how LM proliferation during childhood is different from that in adults and that these LMs proliferate at a significant rate.^37^ The common characteristics of these diseases are lymphatic tissue lesions, including osteolytic lesions, thoracic lesions (chylothorax, pleural effusion, pericardial effusion, mediastinal mass, and retroperitoneal soft-tissue mass), abdominal lesions (ascites, splenic lesions, and retroperitoneal mass), and skin lesions (lymphedema and lymphorrhea). However, to achieve a precise diagnosis, a variety of assessments, clinical, imaging, histological, and hematological examinations, are needed.^2,38^ In terms of the clinical laboratory findings, CLA patients often have hypoalbuminemia, hypoproteinemia, hypogammaglobulinemia, anemia, and lymphocytopenia caused by lymph leakage (chylothorax, pleural effusion, and ascites). They also have moderate or mild thrombocytopenia, low fibrinogen, and coagulopathy (increasing D-dimer and fibrinogen degradation products), called chronic localized intravascular coagulopathy (LIC).^1,2,39^ In general, vascular malformations with slow flow are associated with risk of LIC and deep vein thrombosis. The mechanisms of coagulopathy in CLAs are unknown, but are considered that platelets are involved by abnormal lymphatic endothelium entrapping. The Kasabach–Merritt phenomenon (KMP), which leads to more severe coagulopathy and thrombocytopenia, is associated with vascular tumors, tufted angioma, and kaposiform hemangioendothelioma (KHE). KLA patients also have more severe LIC and thrombocytopenia (50,000–100,000/μL) than those with LM and GLA. A recent study also showed that the serum angiopoietin 2 level in KLA and KHE with KMP patients was higher than in controls.^40^ Therefore, KLA and KHE with KMP may have similar phenotypes and pathogeneses associated with the presence of abnormal spindle cells.

**Table 2.** Characteristics of complex lymphatic anomalies and their differential features

*Characteristics of Complex Lymphatic Anomalies*			
	*GLA/KLA*	*GSD*	*Channel type*
Other terms	Lymphangiomatosis, pulmonary lymphangiomatosis, diffuse pulmonary lymphangiomatosis	Gorham–Stout syndrome, Gorham's disease, vanishing bone disease, massive osteolysis	CCLA, PIL, and pulmonary lymphangiectasia
Definition	The lymphatic anomaly of diffuse or multicentric proliferative lesions as an aggressive disease of the lymphatic system	Progressive destruction and resorption slowly or rapidly. The presence of lymphatics or blood vessels in areas adjacent to the osteolytic bone	Dilated lymphatic channels, distal obstruction affecting lymphatic drainage, and lymphatic channel dysmotility
Clinical features	Multiple organ involvement (mediastinum, lungs, bone, spleen, and soft tissues) involving pleural and pericardial effusion, ascites, mediastinal mass, multiple cystic splenic lesions, gastrointestinal hemorrhage, multiple bone osteolysis, lymphedema, skin involvement, and lymphorrhea	Osteolysis of any bone (skull, maxillofacial skeleton, ribs, pelvis, spine, arms, and legs) progressively and destructively. Cortical bone resorption. Pathological fracture, pain, chronic lymphedema, lymphorrhea, asymmetric girth, joint abnormalities, leg length discrepancy, and scoliosis. Pleural effusion caused by osteolysis of rib bones, thoracic vertebrae, and clavicle	Chylothorax, pulmonary lymphangiectasia, or protein-losing enteropathy. It depends on the affected sites of the anomaly. The etiology is poorly understood. These diseases have a structural abnormality of lymphatic pathological processes

*Differential Features from Other Diseases*	
*GLA vs. KLA*	*GLA/KLA vs. GSD*
• KLA patients have more serious symptoms (thrombocytopenia and hemorrhagic pleural effusion) and a more unfavorable prognosis than those with GLA. • Both diseases show dilated, malformed lymphatic channels, but KLA partially shows foci of patternless clusters of spindle cells. • Ang-2, Ang-2/Ang-1: KLA >controls, GLA	• GLA/KLA: multiple, lytic, and nonprogressive GSD: progressive, infiltrative, and the loss of cortical bone. • GSD: appendicular skeleton, ribs, cranium, clavicle, and cervical spine; GLA: spine, skull • Bone fracture and soft-tissue infiltration surrounding osteolytic lesion: GSD >GLA/KLA • GSD: increasing serum BAP, CTX-1, IL-6, RANKL, OPG/free RANKL

Ang, angiopoietin; BAP, bone-specific alkaline phosphatase; CTX-1, C-terminal telopeptide of type I collagen; IL, interleukin; OPG, osteoprotegerin; PIL, primary intestinal lymphangiectasia; RANKL, ligand for receptor activator of nuclear factor κB.

There is a need to rule out other osteolytic diseases in the differential diagnosis of GSD. Idiopathic multicentric osteolysis, multicentric osteolysis, hereditary multicentric osteolysis, neurogenic osteolysis, and osteolysis due to neoplastic, infectious, and immunological diseases should be considered before reaching a final diagnosis. A series of laboratory investigations were carried out, but they failed to detect osteolysis secondary to pathology (cyst, tumor, and infection), metabolic and endocrinal disorders, neoplastic disease, trauma, and/or connective tissue disorders. A recent case report showed the elevation of bone-specific alkaline phosphatase (BAP) and serum CTX-1 in a GSD patient; the patient showed rapidly decreasing levels of BAP and CTX-1 after bisphosphonate therapy.^41^ Another report showed the elevation of serum tartrate-resistant acid phosphatase before bisphosphonate therapy.^42^ At initial assessment, the elevation of serum IL-6 was found, but the level of IL-6 normalized after 6 months of bisphosphonate treatment. In contrast, the levels of the osteoclastic regulators OPG, free soluble RANKL, and OPG/free RANKL ratio were increased. In GLA and KLA patients, serum angiopoietin 1 and 2 levels were found to be useful for their diagnosis and suitable as biomarkers for drug treatment.^40^ In terms of new radiological methods, Papadakis reported that ^18^F sodium fluoride positron emission tomography/computed tomography (CT), which is used for detecting skeletal metastatic disease, is useful for GSD diagnosis and follow-up.^43^ However, the findings obtained by these methods are only speculative, preventing definitive conclusions from being drawn.

**Figure 5.**
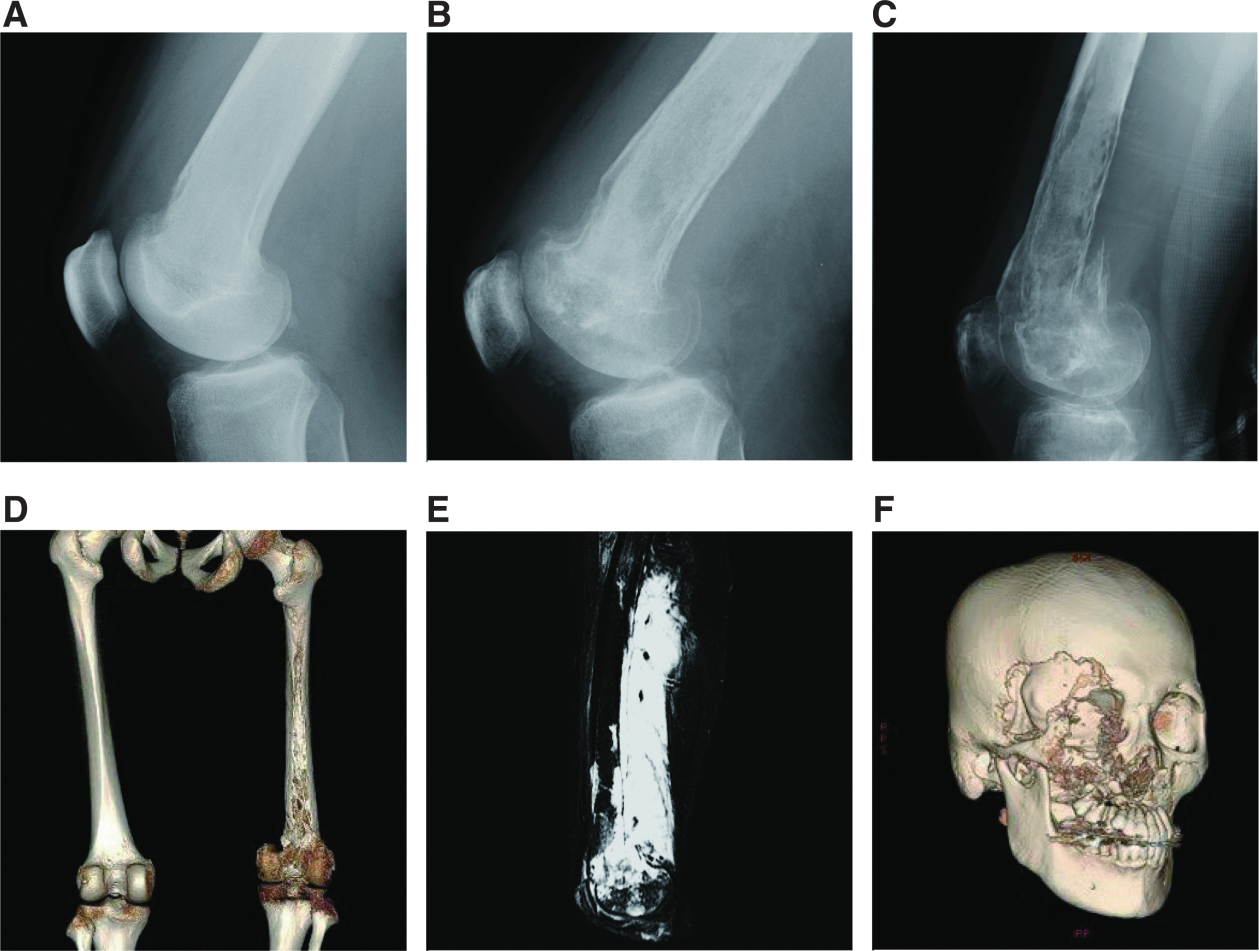
Radiological findings of patients with GSD. The osteolytic imaging of GSD patients is characterized by progressive, destructive cortical resorption that can extend beyond adjacent joints. **(A–E)** A 19-year-old male with GSD involving the right femur. The patient suffered from pain of the right femur, for which no specific cause was evident. **(A)** At onset, a plain radiograph showed osteolysis at the front of the neck of the femur. **(B)** Three months later, the osteolytic lesion had progressed markedly. **(C–E)** Three more months later, the lesion showed pathological fractures. Almost complete resorption of the right femur is shown in posterior view of three-dimensional CT imaging. High signal intensity in the soft tissue surrounding the femur was shown in sagittal fat-saturated T2-weighted MRI, as well as subcutaneous fat tissue. **(F)** A 41-year-old male with GSD involving the right maxillofacial region in the maxilla, the frontal bone, the temporal bone, and the zygomatic bone. The patient complained of sudden vertigo, dysphagia, and left-sided paresthesia. The patient developed skull base osteomyelitis; lateral medullary syndrome resulted from progressive osteolysis and the patient died of associated brainstem stroke. CT, computed tomography; MRI, magnetic resonance imaging.

**Figure 6.**
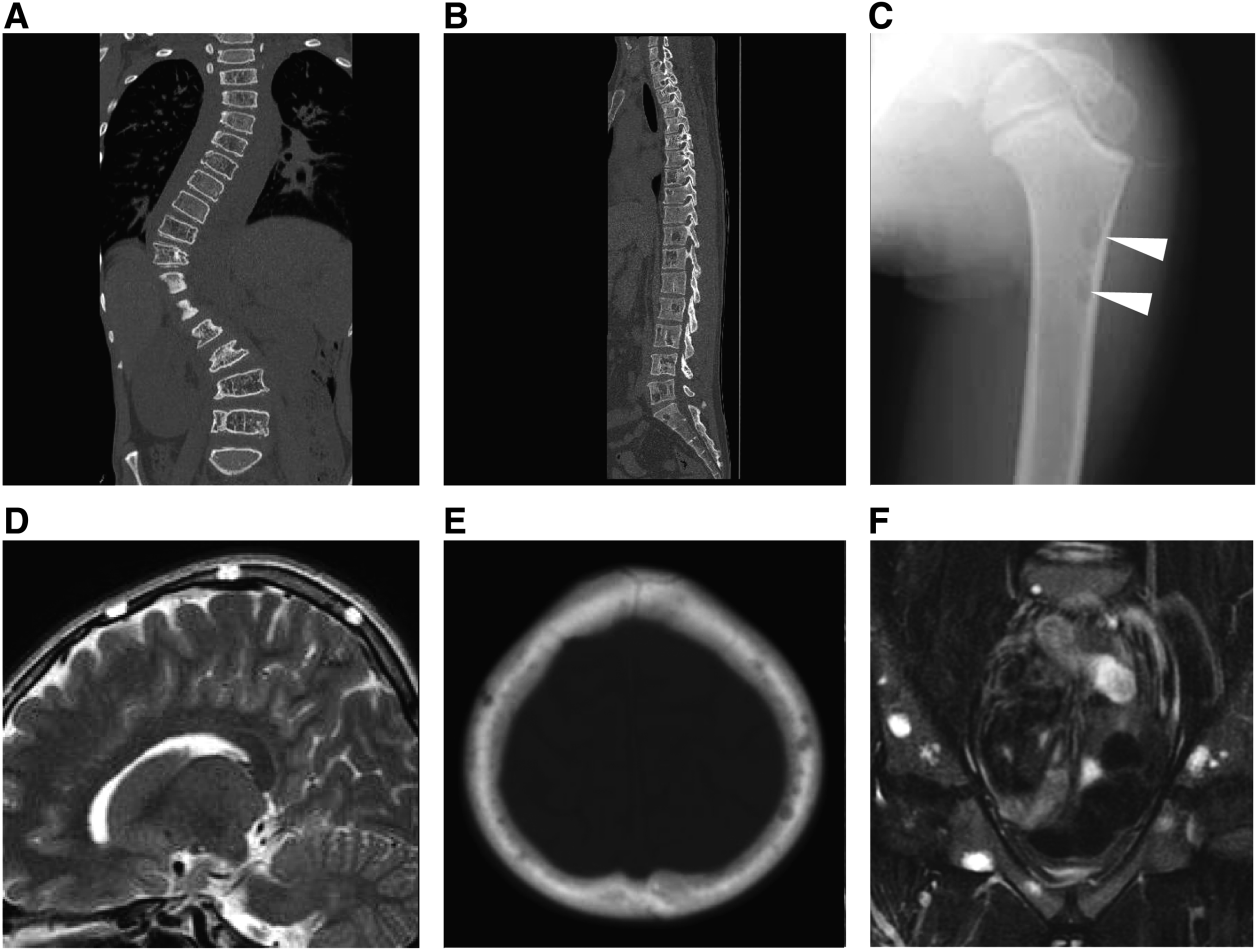
Radiological findings of patients with GLA and KLA. The multiple lytic areas in the medullary cavity are shown in the osteolytic images of GLA and KLA. **(A)** A 12-year-old male with KLA involving the region from the thoracic to lumbar spine. Disseminated osteolysis is shown in CT with coronal reconstruction of in the vertebrae. The patient complained of severe pain, which was treated with opioid. It was difficult to operate to treat his scoliosis because of severe thrombocytopenia and bone fragility. **(B)** A 31-year-old male with GLA involving the region from the thoracic to lumbar spine. He did not complain of any symptoms. **(C–F)** An 8-year-old male with KLA involving multiple bone lesions. He did not also complain of any symptoms. **(C)** A small lytic lesion is shown in plain radiography of femoral head (*white arrowheads*). **(D, E)** Sagittal and axial T2-weighted MRI and CT scans of the head show a small lytic lesion. **(F)** Multiple cystic lesions are shown in a coronal fat-saturated T2-weighted MRI of the pelvis. KLA, kaposiform lymphangiomatosis.

Although there are some overlapping features, the radiological findings in the osteolytic lesion of GSD patients are different from those of GLA/KLA patients. In patients with GLA/KLA, the medullary cavity of osteolytic area shows lytic areas. In contrast, GSD lesions are characterized by progressive osteolysis and the loss of cortical bone^19^ (Figs. 5 and 6), which can extend beyond adjacent joints. The number of affected bone lesions in GLA/KLA is also typically higher compared with GSD. The osteolytic lesion of GSD is commonly localized to the cranium, ribs, clavicle, and cervical spine. Destructive osteolytic lesions can cause pathological fractures, pain, and swelling. However, small multiple lytic lesions might not cause pain in GLA patients (Fig. 6). The GSD patient often has progressive and infiltrative osteolytic lesions (Fig. 5); in contrast, osteolytic lesions of patients with GLA/KLA are not progressive. Furthermore, GLA and KLA patients have vertebral involvement more frequently than GSD patients.^44^ The lumbar spine is the most commonly affected site in GLA and KLA patients.

**Figure 7.**
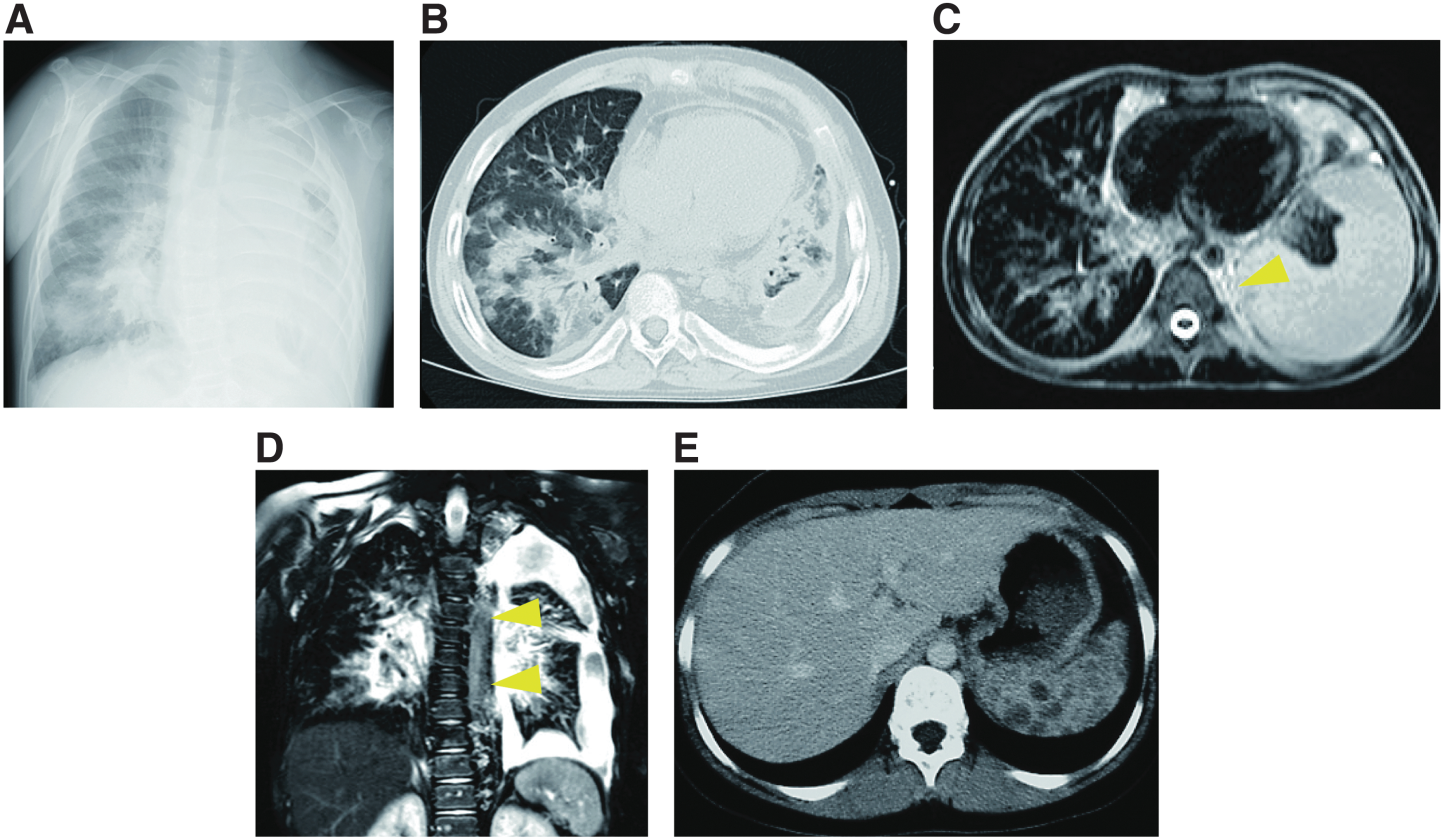
Radiological images of visceral lesions in KLA patients. KLA patients often have mediastinal masses and hemorrhagic pericardial and pleural effusion. In radiological examination, thickening of bronchovascular bundles, retroperitoneal infiltrative soft-tissue thickening, and interlobular septa are common findings. **(A–E)** A 10-year-old male with KLA. He had a mediastinal mass, hemorrhagic pericardial and pleural effusion, and severe coagulopathy. **(A)** Plain radiograph of the chest showed left pleural effusion. **(B)** CT (lung window) showing pleural effusion and bilateral thickening of bronchovascular bundles. **(C, D)** Thoracic T2-weighted MRI in axial and coronal views shows extensive heterogeneous mass (*yellow arrowheads*) images on the left side extending and involving the bronchopulmonary tree and lung parenchyma. **(E)** Abdominal CT demonstrating multiple lesions in the spleen.

In our previous article, we reported the characteristics of GLA, KLA, and GSD.^20^ The following symptoms were more common in GLA/KLA than in GSD: pleural effusion, mediastinal mass, and cardiac effusion. Pleural and cardiac effusion in KLA were similar to that in GLA; KLA patients more frequently have hemorrhagic pericardial and pleural effusions and mediastinal masses than GLA patients. A deteriorating clinical course, retroperitoneal infiltrative soft-tissue thickening, hemorrhagic effusions,, and associated thrombocytopenia are also characteristic of KLA patients.^45^ The mortality in KLA is significantly poorer than GLA (mortality rates: KLA, 55.6%, 5/9; GLA, 28.6%, 10/35, *p* = 0.0268).^20^ Mortality was associated with progressive pulmonary disorders and serious coagulation disorder. The involvement of shoulder girdle or thoracic vertebral bone is predominantly associated with pleural effusion. KLA patient has thickening of bronchovascular bundles and interlobular septa more than GLA^46^ (Fig. 7).

**Figure 8.**
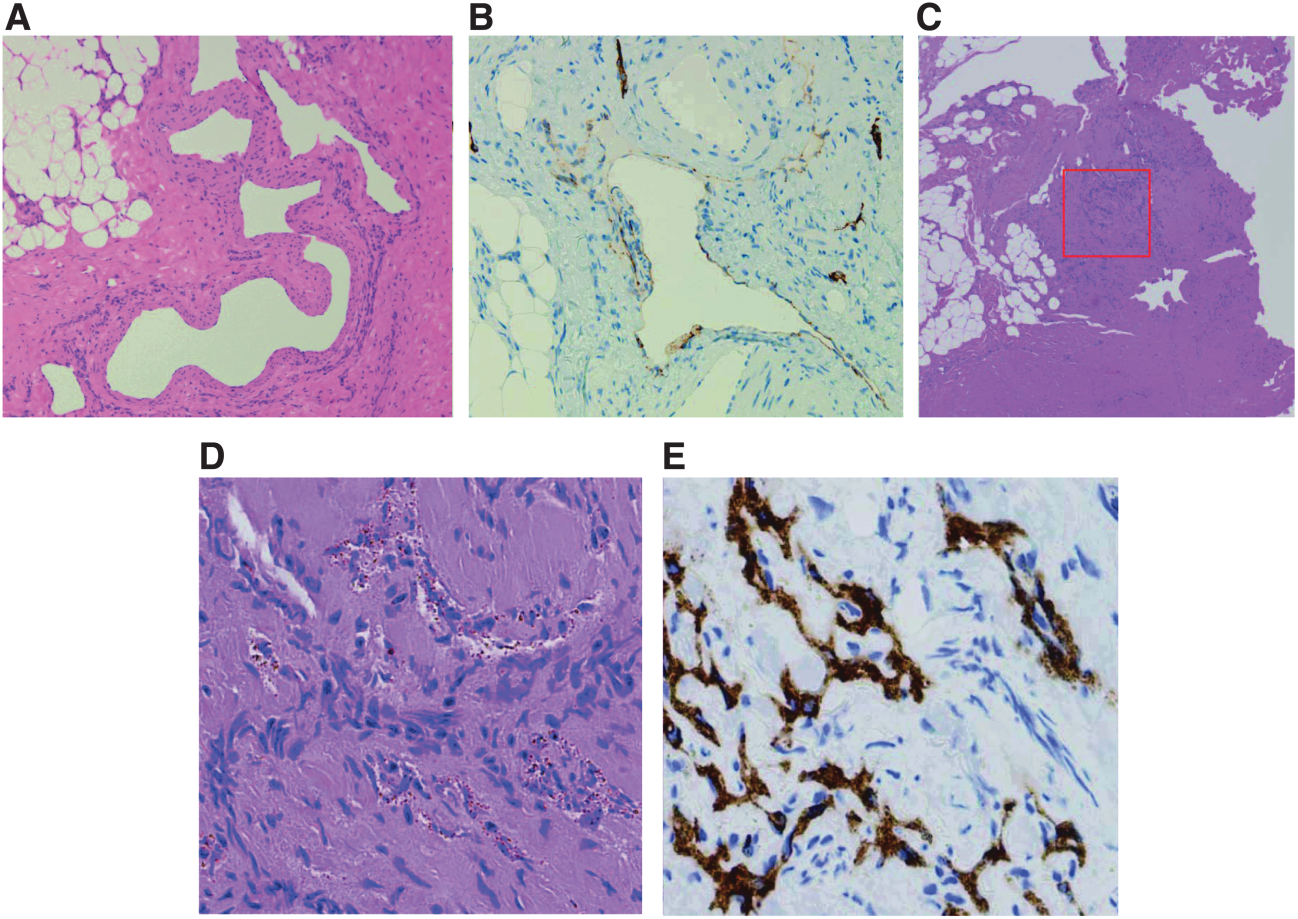
Pathological findings in patients with KLA. In GLA and KLA patients, the typical pathological examinations show dilated malformed lymphatic channels However, foci of patternless clusters of lymphatic spindle cells are characteristic of the patients with KLA. This is associated with, platelet microthrombi, and some degree of fibrosis. **(A–E)** A 20-year-old male with KLA. He had a lymphatic mass lesion of the left chest wall, mediastinal mass, hemorrhagic pleural effusion, severe coagulopathy, and bleeding of the lower digestive tract. A specimen from the subcutaneous lesion was analyzed. **(A)** The proliferation of anastomosing lymphatic vessels without foci of spindle endothelial cells is shown in this specimen (H&E stain). **(B)** Immunostaining of D2-40 is positive. **(C, D)** The proliferation of lymphatic vessels with a focus of spindle cells is shown. Spindle cells can be seen in (**D)** (*red square* in **C**). **(E)** Spindle cells were also positive by D2-40.

If the clinician cannot diagnose the disease by radiological examinations, biopsy of the lesion is the next diagnostic step. Both GLA and KLA show dilated malformed lymphatic channels, but KLA partially shows foci of patternless clusters of spindle cells^20^ (Fig. 8). However, it is not necessary to biopsy all GLAs to rule out KLA. The key features of KLA are thrombocytopenia, coagulation disorder, and hemorrhagic pericardial and pleural effusion or ascites. The presence of an atypical or aggressive pattern can be used to rule out lymphangiosarcoma or other diseases. KHE also features spindle cells and abnormal lymphatic channels, and we need to distinguish KHE or KLA based on the clinical symptoms and distribution of the lesions.^47^ Biopsies of bone tissue specimens are small and often crushed; so poor samples may lead to misdiagnosis. Some articles recommend not performing a rib biopsy because of the high risk of the subsequent development of refractory pleural effusions.^48^ If the lesion spreads superficially, a biopsy of the surface should be performed, although it may not be easy to stop lymph leakage. Idiopathic chylothorax and chylous ascites, which are hard to treat, occur without any invasive examination or treatment. Therefore, if such leakage into the closed lumen is observed, the biopsy can be performed from the lesion on the surface of the thoracic or abdominal cavity.

### Patient assessment, monitoring, and follow-up

Because CLA patients have variable symptoms and problems, they should be consulted by a multidisciplinary team of specialists. Physical assessments and monitoring are very important for overall assessment, monitoring, and follow-up. However, standardized assessment methods for patients with lymphatic anomalies have not yet been established. In general, the following assessment procedures are used: clinical symptoms, radiological evaluation, the amount of effusion, and quality-of-life scale. The clinician should consider the methods used and frequency of examination by condition of the patient and affected lesion(s). If symptoms suggestive of bone lesions are present, patients should undergo screening of the whole body, involving both a skeletal survey and a bone scan. In addition, patients should be evaluated using not only plain radiography but also ultrasound, CT, and magnetic resonance imaging (MRI) to detect adjacent soft-tissue lesions. If no bone lesions are detected, frequent monitoring is not required. GSD patients have progressive bone resorption, so we recommend monitoring by plain radiography of the target bone lesion at least once every 2 months. In contrast, GLA and KLA patients have slowly progressive bone lesions, so we recommend monitoring by plain radiography of the target bone lesion annually. We should also conduct a skeletal survey, as the bone lesion may spread to other parts. Fat-saturated T2-weighted MRI can detect minimal lymphatic lesions (for example, skull bone lesions in GLA patients) that are difficult to detect on plain radiography.

Patients are evaluated using imaging such as ultrasound, CT, and MRI for visceral or adjacent soft-tissue lesions. Lymphoscintigraphy is rapid and minimally invasive without major complications compared with lymphangiography. However, lymphangiography is superior in being able to show detailed images, such as detecting the exact site of leakage of the chylothorax.^2,48^ Regarding the diagnosis of thoracic CLAs, such imaging at an early stage is recommended to be performed to detect the leakage-causing lesion because it can help to obtain not only a precise diagnosis but is also useful information for treatment selection.^49^ A new method, DCMRL, was shown to distinguish central conducting lymphatic dilation from normal tissue of retroperitoneal, which can give us helpful information to diagnose CCLA.^50,51^ The ligation or occlusion of dilated central conducting lymphatics may be an effective therapy for the patients with intractable pleural effusions or ascites.

### Dysfunction of musculoskeletal system and wounds

CLAs involve disorders of the musculoskeletal system and skin, and patients with CLAs sometimes have trouble with wounds. GSD patients often exhibit osteolytic lesions of the appendicular skeleton and have infiltrative soft-tissue lesions surrounding the osteolytic lesion. Soft-tissue lesions show high signal intensity at fat-saturated T2-weighted MRI and demonstrate intense enhancement following the administration of gadolinium contrast agent. These lesions can lead to chronic pain, lymphedema, lymphorrhea, joint abnormalities, asymmetric girth, leg length discrepancy, and scoliosis. Chronic lymphedema or lymphorrhea leads to refractory bleeding, chronic infection, and dermal hypertrophy. Cases with severe cutaneous involvement can exhibit chronic ulceration and angiosarcoma (Stewart–Treves syndrome).^52^ In addition, 35% of KLA patients have subcutaneous masses that are soft, are not tender or painful, and do not grow.^8^ These patients undergo resection, but care should be taken regarding the possibility of local lymphorrhea in the resected area. In such cases, it can sometimes be difficult to stop the leakage. These patients should undergo consultations with specialists such as orthopedists, dermatologists, and plastic surgeons in combination as early as possible.

### Treatment

Clinicians should treat CLA patients corresponding to their condition, complications, and the size and location of the lesion(s). Surgery and interventional therapy are performed in patients with lymphatic anomalies. However, drug treatments are limited and prospective clinical trials are lacking. It is difficult to cure extensive or complicated lesions using only local therapy, so multimodal therapy is necessary. We should begin with conservative treatments (for example, nutrition therapy) if the condition of the patient is not life threatening. The administration of albumin/immunoglobulin and blood transfusion may also be required depending on the patient's symptoms. Aggressive treatment, including medical and surgical treatment, is necessary for cases of more than moderate severity. Bisphosphonates that inhibit bone resorption are used for the treatment of patients with osteolysis.^53^ Meanwhile, interferon, which inhibits the proliferation of blood and lymphatic vessels, is used for patients with bone or generalized lymphatic lesions.^54^ Other pharmaceuticals include the anti-VEGF-A antibody bevacizumab,^55^ propranolol,^56^ steroids, vitamin D, and calcitonin. Although these drugs, in single or combinatorial use, can improve conditions of some patients, effectiveness is limited.

**Figure 9.**
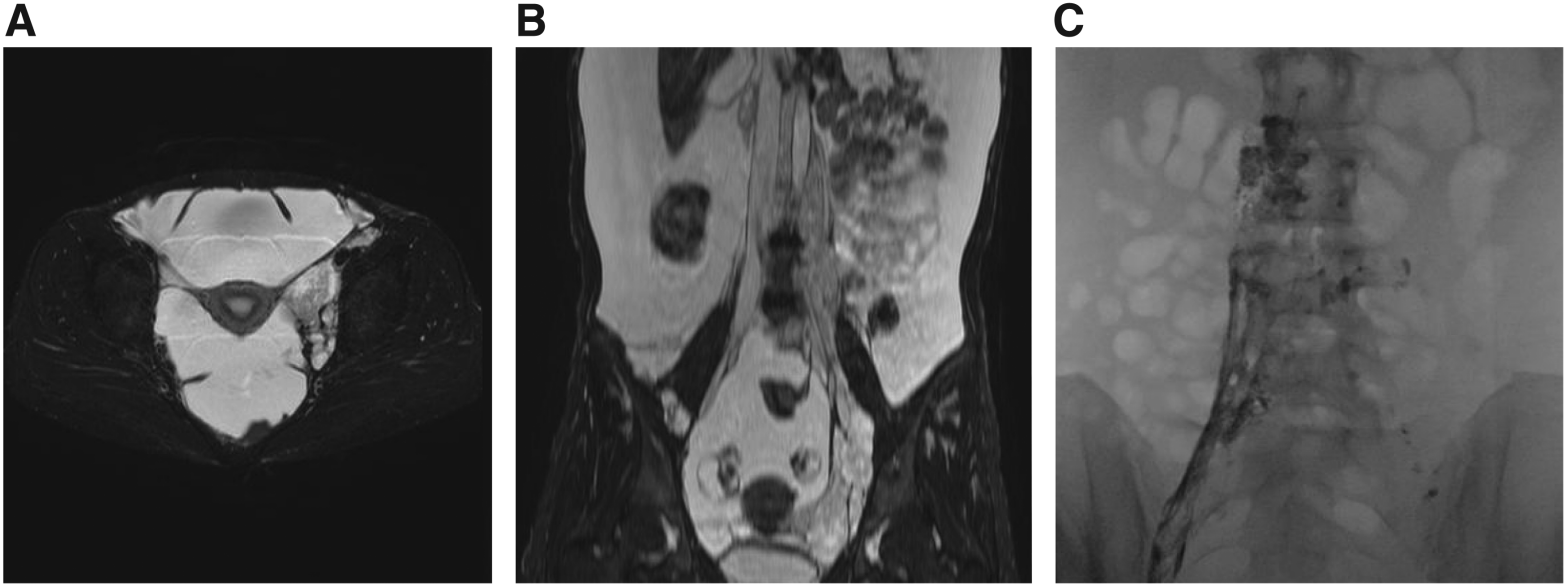
The radiological findings in patients with GLA before sirolimus treatment. **(A–C)** A 32-year-old woman with GLA showing massive ascites and pelvic LM lesions. **(A)** An axial view of T2-weighted MRI showing the left LM lesion adjacent to the uterus. **(B)** An extensive heterogeneous soft mass on the left side extending from the retroperitoneal area to the bladder is shown in a coronal view of T2-weighted MRI. **(C)** A lymphangiography, spot radiograph of the bilateral groin nodes showing the initial phase of intranodal injection of lipiodol with opacification of the bilateral inguinal lymph nodes. The left side shows inadequate lymphatic flow and leakage of lipiodol to the left abdominal cavity from the lymphatic mass lesion.

**Figure 10.**
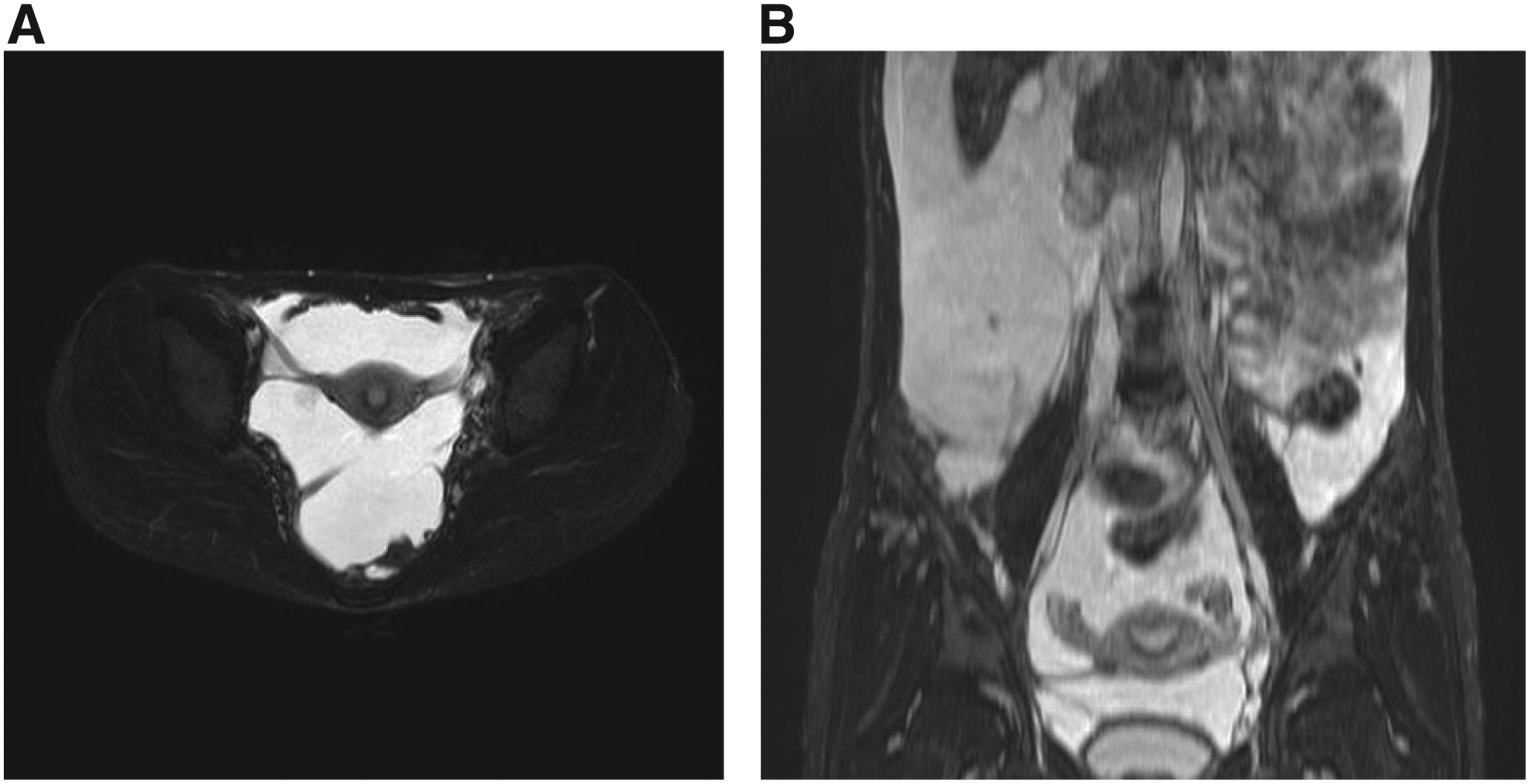
The radiological findings in patients with GLA after sirolimus treatment. **(A, B)** Six months after sirolimus treatment, the mass in the GLA patient was significantly reduced and ascites were also decreased.

In recent studies, the role of the PIK3/AKT/mTOR pathway has been found in the development of blood vessels and lymphatic tissues, and new therapeutic agents that target this pathway are under development. The mTOR inhibitor sirolimus is known to inhibit lymphangiogenesis, and is thought to act on lymphatic tissues within lesions to regulate the production and leakage of lymph by decreasing lymphatic endothelial cell activity^57–60^ (Figs. 9 and 10). In lymphangiectasia model mice, Baluk *et al.* demonstrated that sirolimus inhibited the growth of abnormal lymphatics and reduced lesions without adverse effects on normal lymphatics.^61^ In these treated lymphatic endothelial cells, the expression of Prox1 and VEGFR-3 decreased without the caspase-dependent apoptosis. A prospective trial by Adams *et al.* also indicated that sirolimus had an excellent effect on vascular anomalies, with a good response observed in CLA patients.^60^ This trial reported three distinct assessments involving radiological examination, functional impairment score, and quality of life of the patients. Notably, 100% (7/7) of patients with GLA, 100% (3/3) of patients with GSD, and 71% (5/7) of patients with KLA showed a partial response at 6 months.

The optimal dose of sirolimus remains controversial. Currently, there are no standardized methods for its optimal dosing. A previous study demonstrated that the highest dose of rapamycin had an effect of reducing the incidence of lymphangiectasia, but it might increase toxicities.^61^ A systematic review of sirolimus treatment for vascular anomalies also reported that the expected trough levels of sirolimus for most studies (19/25, 76.0%) were 5–15 ng/mL.^62^ Although these diseases often occur in childhood, data for pediatric patients are very limited. However, population modeling study of sirolimus and recommended dosing for infants was reported by the pharmacological group of Cincinnati Children's Hospital Medical Center.^63–65^ Notably, some articles reported the potential risk of severe side effects (including *Pneumocystis carinii* pneumonia and other infections) in infants treated with sirolimus.^66,67^ When pediatric patients are treated with sirolimus, we should therefore simultaneously perform *Pneumocystis* prophylaxis and monitor the trough levels for safe use. Further clinical trials are important to establish the optimal dosing method of sirolimus, although at present, many prospective clinical trials are ongoing. For example, we are currently undertaking a single-arm, multicenter, prospective study on sirolimus treatment for intractable lymphatic anomalies (SILA study) (UMIN000028905).

In 2018, Venot *et al.* demonstrated that a PIK3CA inhibitor potentially improved the symptoms of patients with PROS.^68^ This was the first evidence suggesting that PIK3CA inhibition is a promising therapeutic strategy in such patients. It is anticipated that the development of such targeted therapies will accelerate in the future.

**Figure 11.**
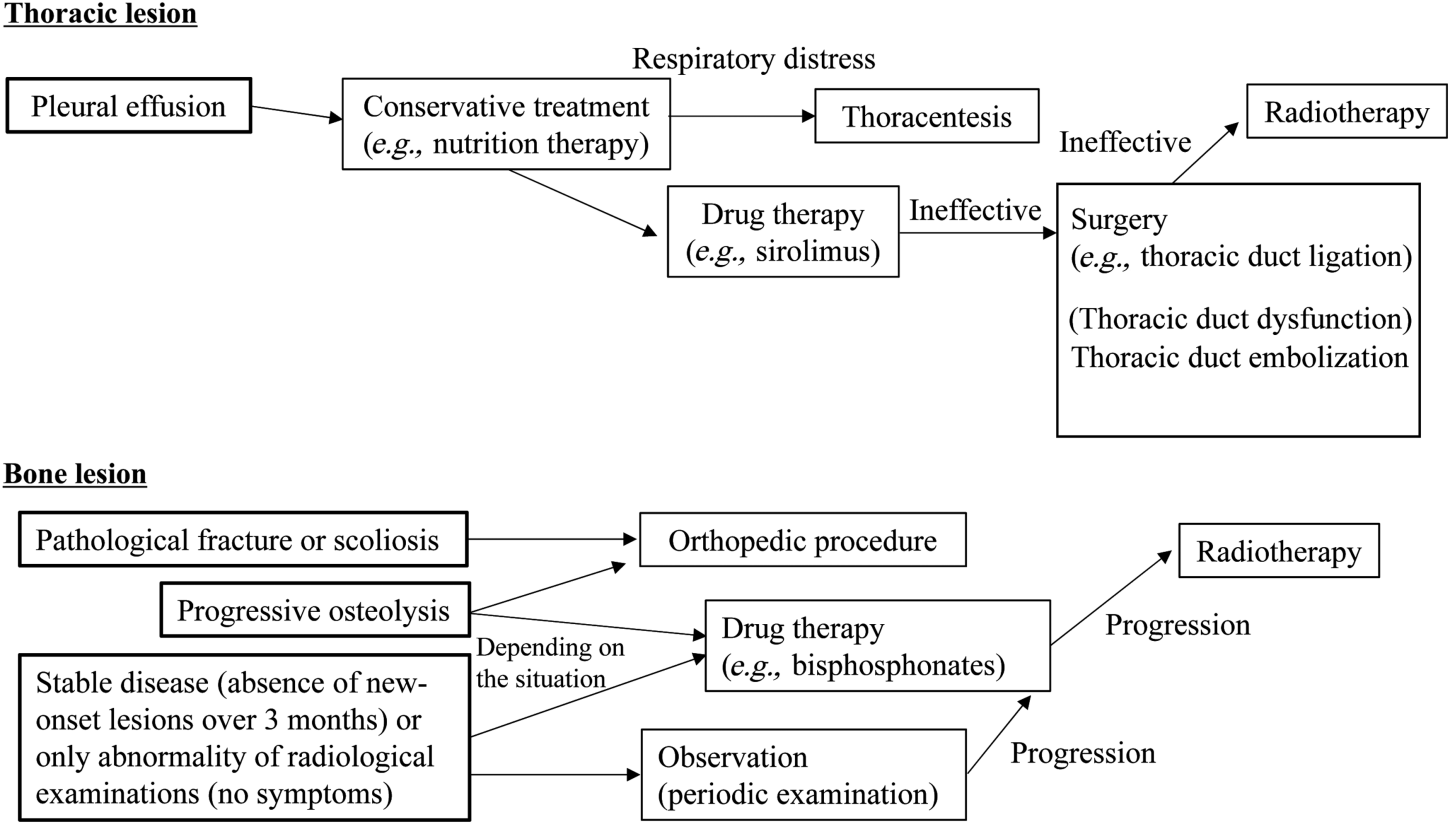
Flow chart of the treatment algorithm for lymphatic anomalies. This flow chart shows the course of treatment for thoracic and bone lesions of lymphatic anomalies. These are only examples and appropriate treatment consistent with individual conditions is necessary.

If pharmacotherapy fails for patients, surgical treatment or radiotherapy should be considered (Fig. 11). The patient with chylothorax undergoes thoracentesis and pleural drainage to improve respiratory distress. If conservative treatment fails, thoracic duct ligation may be effective for pleural effusion. If lymphangiography or DCMRL demonstrates thoracic duct dysfunction, thoracic duct embolization may have a role in improving the patient's status.^69^ Lymphaticovenous anastomosis might also be a therapeutic option for intractable lymphatic pleural effusion or ascites.^70^

If surgery is not possible, radiotherapy is an alternative treatment, independently or simultaneously. A previous study showed that radiation therapy had excellent efficacy in stabilizing disease in ∼80% of GSD patients.^71^ Dunbar *et al.* concluded the patients who were treated with intermediate dose of 40–45 Gy with fractionated radiotherapy.^72^ However, these doses can be associated with the risk of heart and lung radiation hazards due to radiation exposure to the chest. It is known that doses in the 36–45 Gy range have a reliable effect, but further lower doses (16–20 Gy) also appear to be able to relieve thoracic symptoms (chylothorax and chylopericardium). Radiotherapy for pediatric patients may cause other late adverse effects on bone growth disturbance and secondary malignancy; however, cases that do not improve after other treatments cannot avoid radiotherapy to relieve their symptoms. If the patient has lesions of the chest wall pleura and lung parenchyma, they should be treated with radiotherapy of the thorax to prevent crisis in respiratory symptoms.

## Summary

CLAs have extensive and complex lesions, characterized by an abnormal lymphatic system and overlapping clinical symptoms. The patterns of radiological findings of osteolytic lesions can be used to distinguish between GLA/KLA and GSD, along with the presence of infiltrative, progressive bone resorption in GSD patients. In contrast, typical GLA bone lesions are multiple lytic osteolysis. Facilitating the differential diagnosis, KLA patients have a distinctive pathological hallmark, kaposiform hemosiderotic, spindled-shaped lymphatic endothelial cells. Lymphangiography and DCMRL can help to detect abnormalities of the thoracic duct and lymphatic flow in CCLA. Although it remains challenging to diagnose and treat CLAs, the mTOR inhibitor sirolimus could offer a breakthrough for these intractable diseases. In molecular research, data from comprehensive genetic analyses on vascular anomalies allow further insight into their pathogenesis. Pathological animal models also help to deepen our knowledge of the etiology of these diseases. Somatic activating mutations within the genes encoding components of the PIK3/AKT/mTOR and RAS/MAPK pathways have recently been detected in patients with CLAs and numerous vascular anomalies. Identification and characterization of these genetic abnormalities are changing the paradigm by which lesions are diagnosed, potentially leading to novel target therapies and biomarkers.

Take-Home Messages

- CLAs are intractable lymphatic disorders, and include GLA, GSD, KLA, and CCLA. Their pathogenesis is unknown and their differential diagnosis remains difficult due to their similar clinical findings.
- KLA is a subtype of GLA that is characterized by progressive and severe features (severe thrombocytopenia, consumptive coagulopathy, and bleeding), so an accurate diagnosis is vital.
- Osteolytic imaging can distinguish between GLA/KLA and GSD.
- Lymphangiography and DCMRL can facilitate the identification of abnormal structure of lymphatics vessels or channels, which may be responsible for leakage of lymph fluid.
- Sirolimus might have efficacy in improving the symptoms of patients and could be a novel first-line therapy for CLA patients.
- The genes encoding components of the PIK3CA and RAS pathways are associated with the pathology of CLAs, understanding of which can deepen our knowledge of diseases and lead to novel target treatments.

## About the Authors

**Michio Ozeki, MD, PhD**, is a senior lecturer at the Department of Pediatrics, Gifu University. **Toshiyuki Fukao, MD, PhD**, is a professor at the Department of Pediatrics, Gifu University. The authors have researched the epidemiological characteristics of CLAs in Japan. Their research focuses on the etiology and clinical aspects of CLAs. Recent interests include understanding the role of the PIK3/Akt/mTOR pathway in vascular anomalies and target therapies.

## Abbreviations and Acronyms

CCLA: central conducting lymphatic anomaly
CLAs: complex lymphatic anomalies
CLOVES: congenital lipomatous overgrowth, vascular malformations, epidermal nevi, scoliosis/skeletal and spinal
CT: computed tomography
CTX-1: C-terminal telopeptide of type I collagen
DCMRL: dynamic contrast-enhanced magnetic resonance lymphangiography
EFNB2: ephrin B2
EPHB4: ephrin B4
GLA: generalized lymphatic anomaly
GSD: Gorham–Stout disease
IL: interleukin
ISSVA: International Society for the Study of Vascular Anomalies
KHE: kaposiform hemangioendothelioma
KLA: kaposiform lymphangiomatosis
KMP: Kasabach–Merritt phenomenon
LIC: localized intravascular coagulopathy
LM: lymphatic malformation
LyEC: lymphangiomatosis endothelial cell
MAPK: mitogen-activated protein kinase
MRI: magnetic resonance imaging
mTOR: mammalian target of rapamycin
OPG: osteoprotegerin
PI3K: phosphoinositide 3-kinase
PIK3CA: phosphatidylinositol-4,5-bisphosphate 3-kinase catalytic subunit alpha
PROS: PIK3CA-related overgrowth spectrum
RANK: receptor activator of nuclear factor κB
RANKL: ligand for receptor activator of nuclear factor κB
VEGF: vascular endothelial growth factor
VEGFR: VEGF receptor

## References

[1] International Society for the Study of Vascular Anomalies: ISSVA classification for Vascular Anomalies (approved at the May 2018 General Assembly in Amsterdam, the Netherlands). http://issva.org/classification (last accessed 62018)

[2] Trenor CC 3rd, Chaudry G Complex lymphatic anomalies. Semin Pediatr Surg 2014;23:186–190 25241096 10.1053/j.sempedsurg.2014.07.006

[3] Blei F Lymphangiomatosis: clinical overview. Lymphat Res Biol 2011;9:185–190 22196283 10.1089/lrb.2011.0020

[4] Radhakrishnan K, Rockson SG Gorham's disease: an osseous disease of lymphangiogenesis? Ann NY Acad Sci 2008;1131:203–205 18519972 10.1196/annals.1413.022

[5] Michael TD, Nupur G, Bjorn RO Viewpoints on vessels and vanishing bones in Gorham–Stout disease. Bone 2014;63:47–52 24583233 10.1016/j.bone.2014.02.011

[6] Croteau SE, Kozakewich HP, Perez-Atayde AR, et al. Kaposiform lymphangiomatosis: a distinct aggressive lymphatic anomaly. J Pediatr 2014;164:383–388 24252784 10.1016/j.jpeds.2013.10.013 PMC3946828

[7] Safi F, Gupta A, Adams D, Anandan V, McCormack FX, Assaly R Kaposiform lymphangiomatosis, a newly characterized vascular anomaly presenting with hemoptysis in an adult woman. Ann Am Thorac Soc 2014;11:92–95 24460439 10.1513/AnnalsATS.201308-287BC

[8] Fernandes VM, Fargo JH, Saini S, et al. Kaposiform lymphangiomatosis: unifying features of a heterogeneous disorder. Pediatr Blood Cancer 2015;62:901–904 25307772 10.1002/pbc.25278

[9] Faul JL, Berry GJ, Colby TV, et al. Thoracic lymphangiomas, lymphangiectasis, lymphangiomatosis, and lymphatic dysplasia syndrome. Am J Respir Crit Care Med 2000;161:1037–1046 10712360 10.1164/ajrccm.161.3.9904056

[10] Tammela T, Saaristo A, Holopainen T, et al. Therapeutic differentiation and maturation of lymphatic vessels after lymph node dissection and transplantation. Nat Med 2007;13:1458–1466 18059280 10.1038/nm1689

[11] Yao LC, Testini C, Tvorogov D, et al. Pulmonary lymphangiectasia resulting from vascular endothelial growth factor-C overexpression during a critical period. Circ Res 2014;114:806–822 24429550 10.1161/CIRCRESAHA.114.303119 PMC3969887

[12] Nitschké M, Bell A, Karaman S, et al. Retrograde lymph flow leads to chylothorax in transgenic mice with lymphatic malformations. Am J Pathol 2017;187:1984–1997 28683257 10.1016/j.ajpath.2017.05.009 PMC5808174

[13] Hominick D, Silva A, Khurana N, et al. VEGF-C promotes the development of lymphatics in bone and bone loss. Elife 2018;7:e34323 29620526 10.7554/eLife.34323 PMC5903859

[14] Greene AK, Goss JA Vascular anomalies: from a clinicohistologic to a genetic framework. Plast Reconstr Surg 2018;141:709e–717e 10.1097/PRS.0000000000004294 PMC5922803 29697621

[15] Brouillard P, Boon L, Vikkula M Genetics of lymphatic anomalies. J Clin Invest 2014;124:898–904 24590274 10.1172/JCI71614 PMC3938256

[16] Osborn AJ, Dickie P, Neilson DE, et al. Activating PIK3CA alleles and lymphangiogenic phenotype of lymphatic endothelial cells isolated from lymphatic malformations. Hum Mol Genet 2015;24:926–938 25292196 10.1093/hmg/ddu505

[17] Boscolo E, Coma S, Luks VL, et al. AKT hyper-phosphorylation associated with PI3K mutations in lymphatic endothelial cells from a patient with lymphatic malformation. Angiogenesis 2015;18:151–162 25424831 10.1007/s10456-014-9453-2 PMC4366356

[18] Manevitz-Mendelson E, Leichner GS, Barel O, et al. Somatic NRAS mutation in patient with generalized lymphatic anomaly. Angiogenesis 2018;21:287–298 29397482 10.1007/s10456-018-9595-8

[19] Lala S, Mulliken JB, Alomari AI, Fishman SJ, Kozakewich HP, Chaudry G Gorham–Stout disease and generalized lymphatic anomaly–clinical, radiologic, and histologic differentiation. Skeletal Radiol 2013;42:917–924 23371338 10.1007/s00256-012-1565-4

[20] Ozeki M, Fujino A, Matsuoka K, Nosaka S, Kuroda T, Fukao T Clinical features and prognosis of generalized lymphatic anomaly, kaposiform lymphangiomatosis, and Gorham-Stout disease. Pediatr Blood Cancer 2016;63:832–838 26806875 10.1002/pbc.25914

[21] Nadolski G Nontraumatic chylothorax: diagnostic algorithm and treatment options. Tech Vasc Interv Radiol 2016;19:286–290 27993324 10.1053/j.tvir.2016.10.008

[22] Wassef M, Blei F, Adams D, et al.; ISSVA Board and Scientific Committee Vascular anomalies classification: recommendations from the International Society for the Study of Vascular Anomalies. Pediatrics 2015;136:e203–e214 26055853 10.1542/peds.2014-3673

[23] Fletcher CDM, Bridge JA, Hogendoorn PCW, Eds FM Vascular Tumours, Vol. 5. Lyon, France: IARC Publications, 2013:137

[24] Luks VL, Kamitaki N, Vivero MP, et al. Lymphatic and other vascular malformative/overgrowth disorders are caused by somatic mutations in PIK3CA. J Pediatr 2015;166:1048–1054 25681199 10.1016/j.jpeds.2014.12.069 PMC4498659

[25] Keppler-Noreuil KM, Rios JJ, Parker VE, et al. PIK3CA-related overgrowth spectrum (PROS): diagnostic and testing eligibility criteria, differential diagnosis, and evaluation. Am J Med Genet A 2015;167:287–295 10.1002/ajmg.a.36836 PMC4480633 25557259

[26] Khan AQ, Kuttikrishnan S, Siveen KS, et al. RAS-mediated oncogenic signaling pathways in human malignancies. Semin Cancer Biol. 2018 Mar 7. pii: . doi: 10.1016/j.semcancer.2018.03.001. [Epub ahead of print] 29524560

[27] Li D, Wenger TL, Seiler C, et al. Pathogenic variant in EPHB4 results in central conducting lymphatic anomaly. Hum Mol Genet 2018;27:3233–3245 29905864 10.1093/hmg/ddy218 PMC7190898

[28] Mäkinen T, Adams RH, Bailey J, et al. PDZ interaction site in ephrinB2 is required for the remodeling of lymphatic vasculature. Genes Dev 2005;19:397–410 15687262 10.1101/gad.330105 PMC546518

[29] Zhang G, Brady J, Liang WC, Wu Y, Henkemeyer M, Yan M EphB4 forward signalling regulates lymphatic valve development. Nat Commun 2015;6:6625 25865237 10.1038/ncomms7625 PMC4403310

[30] Hagendoorn J, Yock TI, Borel Rinkes IH, Padera TP, Ebb DH Novel molecular pathways in Gorham disease: implications for treatment. Pediatr Blood Cancer 2014;61:401–406 24214028 10.1002/pbc.24832 PMC4123459

[31] Franco-Barrera MJ, Zavala-Cerna MG, Aguilar-Portillo G, et al. Gorham-Stout disease: a clinical case report and immunological mechanisms in bone erosion. Clin Rev Allergy Immunol 2017;52:125–132 28004375 10.1007/s12016-016-8594-z

[32] Lohela M, Bry M, Tammela T, Alitalo K VEGFs and receptors involved in angiogenesis versus lymphangiogenesis. Curr Opin Cell Biol 2009;21:154–165 19230644 10.1016/j.ceb.2008.12.012

[33] Joukov V, Pajusola K, Kaipainen A, et al. A novel vascular endothelial growth factor, VEGF-C, is a ligand for the Flt4 (VEGFR-3) and KDR (VEGFR-2) receptor tyrosine kinases. EMBO J 1996;15:290–298 8617204 PMC449944

[34] Brodszki N, Länsberg JK, Dictor M, et al. A novel treatment approach for paediatric Gorham-Stout syndrome with chylothorax. Acta Paediatr 2011;100:1448–1453 21605166 10.1111/j.1651-2227.2011.02361.x

[35] Garnero P, Ferreras M, Karsdal MA, et al. The type I collagen fragments ICTP and CTX reveal distinct enzymatic pathways of bone collagen degradation. J Bone Miner Res 2003;18:859–867 12733725 10.1359/jbmr.2003.18.5.859

[36] Zavala-Cerna MG, Moran-Moguel MC, Cornejo-Toledo JA, et al. Osteoprotegerin polymorphisms in a Mexican population with rheumatoid arthritis and generalized osteoporosis: a preliminary report. J Immunol Res 2015;2015:376197 26065000 10.1155/2015/376197 PMC4433710

[37] Mori M, Dictor M, Brodszki N, et al. Pulmonary and pleural lymphatic endothelial cells from pediatric, but not adult, patients with Gorham-Stout disease and generalized lymphatic anomaly, show a high proliferation rate. Orphanet J Rare Dis 2016 18;11:67 27194137 10.1186/s13023-016-0449-4 PMC4870727

[38] Adams DM, Fishman SJ Late sequelae and long-term outcomes of vascular anomalies. Semin Pediatr Surg 2017;26:317–321 29110828 10.1053/j.sempedsurg.2017.09.007 PMC13270361

[39] Nakano TA, Zeinati C Venous thromboembolism in pediatric vascular anomalies. Front Pediatr 2017 24;5:158 28791278 10.3389/fped.2017.00158 PMC5522837

[40] Le Cras TD, Mobberley-Schuman PS, Broering M, Fei L, Trenor CC 3rd, Adams DM Angiopoietins as serum biomarkers for lymphatic anomalies. Angiogenesis 2017;20:163–173 27990590 10.1007/s10456-016-9537-2

[41] Yerganyan VV, Body JJ, De Saint Aubain N, Gebhart M Gorham-Stout disease of the proximal fibula treated with radiotherapy and zoledronic acid. J Bone Oncol 2015;16:42–46 10.1016/j.jbo.2015.05.001 PMC4620947 26579487

[42] Hammer F, Kenn W, Wesselmann U, et al. Gorham-Stout disease—stabilization during bisphosphonate treatment. J Bone Miner Res 2005;20:350–353 15647829 10.1359/JBMR.041113

[43] Papadakis GZ, Millo C, Bagci U, Blau J, Collins MT 18F-NaF and 18F-FDG PET/CT in Gorham-Stout disease. Clin Nucl Med 2016;41:884–885 27648707 10.1097/RLU.0000000000001369 PMC5067956

[44] Kato H, Ozeki M, Fukao T, Matsuo M MR imaging findings of vertebral involvement in Gorham-Stout disease, generalized lymphatic anomaly, and kaposiform lymphangiomatosis. Jpn J Radiol 2017;35:606–612 28795277 10.1007/s11604-017-0674-3

[45] Kato H, Ozeki M, Fukao T, Matsuo M Chest imaging in generalized lymphatic anomaly and kaposiform lymphangiomatosis. Pediatr Int 2018. doi: 10.1111/ped.13593. [Epub ahead of print] 29923669

[46] Goyal P, Alomari AI, Kozakewich HP, et al. Imaging features of kaposiform lymphangiomatosis. Pediatr Radiol 2016;46:1282–1290 27053281 10.1007/s00247-016-3611-1

[47] Croteau SE, Liang MG, Kozakewich HP, et al. Kaposiform hemangioendothelioma: atypical features and risks of Kasabach-Merritt phenomenon in 107 referrals. J Pediatr 2013;162:142–147 22871490 10.1016/j.jpeds.2012.06.044 PMC3494787

[48] Adams DM, Brandão LR, Peterman CM, et al. Vascular anomaly cases for the pediatric hematologist oncologists-an interdisciplinary review. Pediatr Blood Cancer 2018;65:e26716 10.1002/pbc.26716 28727248

[49] Ludwig KF, Slone T, Cederberg KB, Silva AT, Dellinger M A new case and review of chylothorax in generalized lymphatic anomaly and Gorham-Stout disease. Lymphology 2016;49:73–84 29906363

[50] Pan HP, Lao Q, Fei ZH, Yang L, Zhou HC, Lai C MR lymphangiography for focal disruption of the thoracic duct in chylothorax of an infant: a case report and literature review. Chin Med Sci J 2017 30;32:265–268 29301603 10.24920/J1001-9294.2017.038

[51] Pimpalwar S, Chinnadurai P, Chau A, et al. Dynamic contrast enhanced magnetic resonance lymphangiography: categorization of imaging findings and correlation with patient management. Eur J Radiol 2018;101:129–135 29571786 10.1016/j.ejrad.2018.02.021

[52] Veiga RR, Nascimento BA, Carvalho AH, Brito AC, Bittencourt Mde J Stewart-Treves Syndrome of the lower extremity. An Bras Dermatol 2015;90:232–234 10.1590/abd1806-4841.20153926 PMC4540560 26312726

[53] Kuriyama DK, McElligott SC, Glaser DW, Thompson KS Treatment of Gorham–Stout disease with zoledronic acid and interferon-alpha: a case report and literature review. J Pediatr Hematol Oncol 2010;32:579–584 20962674 10.1097/MPH.0b013e3181edb464

[54] Ozeki M, Funato M, Kanda K, et al. Clinical improvement of diffuse lymphangiomatosis with pegylated interferon alfa-2b therapy: case report and review of the literature. Pediatr Hematol Oncol 2007;24:513–524 17786787 10.1080/08880010701533603

[55] Grunewald TG, Damke L, Maschan M, et al. First report of effective and feasible treatment of multifocal lymphangiomatosis (Gorham–Stout) with bevacizumab in a child. Ann Oncol 2010;21:1733–1734 20605931 10.1093/annonc/mdq331

[56] Ozeki M, Fukao T, Kondo N Propranolol for intractable diffuse lymphangiomatosis. N Engl J Med 2011;364:1380–1382 21470038 10.1056/NEJMc1013217

[57] Hammill AM, Wentzel M, Gupta A, et al. Sirolimus for the treatment of complicated vascular anomalies in children. Pediatr Blood Cancer 2011;57:1018–1024 21445948 10.1002/pbc.23124

[58] Lackner H, Karastaneva A, Schwinger W, et al. Sirolimus for the treatment of children with various complicated vascular anomalies. Eur J Pediatr 2015;174:1579–1584 26040705 10.1007/s00431-015-2572-y

[59] Reinglas J, Ramphal R, Bromwich M The successful management of diffuse lymphangiomatosis using sirolimus: a case report. Laryngoscope 2011;121:1851–1854 22024836 10.1002/lary.21927

[60] Adams DM, Trenor CC 3rd, Hammill AM, et al. Efficacy and safety of sirolimus in the treatment of complicated vascular anomalies. Pediatrics 2016;137:1–10 10.1542/peds.2015-3257 PMC4732362 26783326

[61] Baluk P, Yao LC, Flores JC, Choi D, Hong YK, McDonald DM Rapamycin reversal of VEGF-C-driven lymphatic anomalies in the respiratory tract. JCI Insight 2017;2:e90103 10.1172/jci.insight.90103 PMC5621869 28814666

[62] Nadal M, Giraudeau B, Tavernier E, et al. Efficacy and safety of mammalian target of rapamycin inhibitors in vascular anomalies: a systematic review. Acta Derm Venereo 2016;96:448–452 10.2340/00015555-2300 26607948

[63] Mizuno T, Emoto C, Fukuda T, Hammill AM, Adams DM, Vinks AA Model-based precision dosing of sirolimus in pediatric patients with vascular anomalies. Eur J Pharm Sci 2017 15;109:124–131 10.1016/j.ejps.2017.05.037 28526601

[64] Mizuno T, Fukuda T, Emoto C, et al. Developmental pharmacokinetics of sirolimus: implications for precision dosing in neonates and infants with complicated vascular anomalies. Pediatr Blood Cancer 2017;64:e26470 10.1002/pbc.26470 28205374

[65] Emoto C, Fukuda T, Mizuno T, et al. Characterizing the developmental trajectory of sirolimus clearance in neonates and infants. CPT Pharmacometrics Syst Pharmacol 2016;5:411–417 27501453 10.1002/psp4.12096 PMC4999604

[66] Ying H, Qiao C, Yang X, Lin X A case report of 2 sirolimus-related deaths among infants with kaposiform hemangioendotheliomas. Pediatrics 2018;141:425–429 10.1542/peds.2016-2919 29610165

[67] Russell TB, Rinker EK, Dillingham CS, Givner LB, McLean TW *Pneumocystis jirovecii* pneumonia during sirolimus therapy for kaposiform hemangioendothelioma. Pediatrics 2018;141:421–424 10.1542/peds.2017-1044 29610164

[68] Venot Q, Blanc T, Rabia SH, et al. Targeted therapy in patients with PIK3CA-related overgrowth syndrome. Nature 2018;558:540–546 29899452 10.1038/s41586-018-0217-9 PMC7610773

[69] Itkin M Interventional treatment of pulmonary lymphatic anomalies. Tech Vasc Interv Radiol 2016;19:299–304 27993326 10.1053/j.tvir.2016.10.005

[70] Mihara M, Hara H, Shibasaki J, et al. Indocyanine green lymphography and lymphaticovenous anastomosis for generalized lymphatic dysplasia with pleural effusion and ascites in neonates. Ann Vasc Surg 2015;29:1111–1122 26025477 10.1016/j.avsg.2015.02.013

[71] Heyd R, Micke O, Surholt C, et al. German Cooperative Group on Radiotherapy for Benign Diseases (GCG-BD). Radiation therapy for Gorham–Stout syndrome: results of a national patterns-of-care study and literature review. Int J Radiat Oncol Biol Phys 2011;81:179–185 10.1016/j.ijrobp.2011.01.006 21345608

[72] Dunbar SF, Rosenberg A, Mankin H, Rosenthal D, Suit HD Gorham's massive osteolysis: the role of radiation therapy and a review of the literature. Int J Radiat Oncol Biol Phys 1993;26:491–497 8514544 10.1016/0360-3016(93)90968-2

